# Porcelain Art. Inlays: Some Suggestions Conducive to Success in the Practice

**Published:** 1904-05

**Authors:** H. W. Arthur

**Affiliations:** Pittsburg, Pa.


THE AMERICAN

### Journal of Dental Science.

### PORCELAIN ART. INLAYS: SOME SUGGESTIONS CONDUCIVE TO SUCCESS IN THE PRACTICE.

By H. W. ARTHUR, D.D.S., Pittsburg, Pa.

Read before the Lake Erie Dental Society.

The highest art is said to be the concealing of art.

The inlay, applied with skill, is the ideal of art in operative dentistry. The use of the porcelain inlay was the outcome of the desire to conceal the ravages of caries. The practice has extended until the practicability of securing a matrix is the only limit to its application.

The time limit will not permit, nor is it necessary at this time to give in detail the methods of making porcelain inlays. These have been exploited so often and so recently, that even those who have not taken up the practice ought to be familiar with the procedures.

To succeed in the practice, each and every step should be conducted with care and exactness. The rules for the preparation of margins should be strictly observed. The outlines of cavities should be in definite curves and straight lines. The margins of cavities should be at right angles with the surfaces of the teeth. The margins should be smooth. This means something more than the ordinary care shown in the preparation of margins for the filling operation. Where cavities are of direct access, the forming of a matrix is not difficult. Proximal cavities, where two or more surfaces are involved, are more complicated. Dr. Reeves's method, the use of wet cotton, for general adaptation to the cavity, linen tape and a strip of rubber dam for margins, is most efficient. Where the cavity is difficult to approach and the space limited, as on proximal surfaces, a perfectly adapted matrix may be distorted in the removal. Exactness is so necessary that a slight distortion not observed, it may be, until the matrix is stripped from the inlay, must be guarded against. An efficient *expedient* in such cases is, after the matrix is fitted and resting in the cavity, in place, to fill the matrix with pure beeswax, not too soft. This should be trimmed to the margins, so that there will be no interference with the removal of the matrix. Beeswax may be also used as an expedient where, after stripping the matrix, the inlay is found defective in some part. A new matrix is adapted to the cavity, the inlay is placed in the matrix, held firmly in place and beeswax rather hard forced into the defective outlines. The beeswax can be readily removed by placing the matrix in the mouth of the muffle. It may be somewhat startling to the novice to see the inlay at first turn black, but every trace of the wax will burn off and disappear. To avoid any possible change in the form of the matrix, it may be invested in asbestos powder before attempting to remove the wax.

Inlays are the severest tests for porcelain shades; no more difficult task presents itself than that of accurate matching. It must be borne in mind that the dry shade of a tooth is not the same as the moist, that the shade matching the labial surface will be dark on the proximal (within the "shadow lines").

The selection of shades in the porcelain filling materials is a problem to be worked out in each particular case. Mass shade is not so perfect, nor is it so readily controlled, as the body and enamel shading. Shade effects are simplified by the use of shaded body for the bisque baking, and by the use of porcelain enamels, combined shades, or separate application; that is, one layer of shade over another, or surface blending.

The swaged matrix, which can be duplicated, affords a means for baking several inlays of different shading, from which a selection can be made for insertion.

A likely change in the appearance of the shade of the inlay when inserted in the oxyphosphate must not be lost sight of. To some extent this may be controlled. An enthusiastic porcelain inlay worker says: "An inlay built up in layers will prevent the reflection of the cement through from beneath." An increase in the number and variety of shades of the oxychloride is desirable.

An inlay when finished should have well defined margins; a feather edge may fracture and leave a ragged, imperfect margin. The time to avoid this is at the final touching up of the porcelain filling material, before placing it in the furnace. A fine camel's hair brush, slightly moistened, should be used, brushing towards the filling, taking away all fine particles.

One thing in the practice of making porcelain inlays has been very much simplified; that is, the fusing of the porcelain materials. The small furnaces, especially the electric, work admirably. A knowledge of the fusing points of porcelain materials once attained, definite results thereafter are assured.

With simple inlays the removal of the glaze from the imbedded side is all that is necessary to insure retention. With contour inlays, while some claim to have no difficulties, such has not been our experience. To overcome a difficulty of this kind in a superior cuspid, mesio-incisal angle to be restored, a resort was made to reducing the contour to a simple cavity, by restoring with gold the lingual and proximal surfaces and the incisal edge; the force of the bite being on the gold. A simple inlay was inserted in the gold, labial surface. In another case, mesial surface of an upper first bicuspid, an inlay, made for the mesial surface, extending beyond the mesial ridge, occlusal surface; the extension of the inlay, along the occlusal surface, was cut down, inside of the mesial ridge; a filling, previously inserted on the occlusal and distal surface, was partially cut out, shaped for retention, and refilled, overlapping the reduced portion of the inlay.

